# 染色体外癌基因扩增特点与肿瘤发生发展

**DOI:** 10.3779/j.issn.1009-3419.2020.101.48

**Published:** 2020-12-20

**Authors:** 雨彤 汪, 凡 叶, 霄 张, 睿涵 邹, 明远 王, 锴 俞, 诗允 崔

**Affiliations:** 1 211166 南京，南京医科大学第一临床医学院 Nanjing Medical University, Nanjing 211166, China; 2 210029 南京，南京医科大学第一附属医院肿瘤科 Department of Oncology, The First Affiliated Hospital of Nanjing Medical University, Nanjing 210029, China

**Keywords:** 染色体外DNA, 双微体, 染色体外环状DNA, 肿瘤, Extrachromosomal DNA, Double minutes, Extrachromosomal circular DNA, Tumor

## Abstract

染色体外DNA（extrachromosomal DNA, ecDNA）是位于染色体外的小片段环状DNA，具有自我复制功能。已有研究证明癌基因在ecDNA上被扩增是肿瘤细胞中的常见现象，并且具有一些值得研究的特征，如与患者的不良预后相关。多种染色体事件参与ecDNA形成，且它的扩增能直接使染色体外癌基因DNA拷贝数增加，加速肿瘤产生和发展。并且，亲代ecDNA细胞不均等传递到子代的分离方式，不仅增加肿瘤异质性，同时增强肿瘤对环境的适应和对治疗的反应能力。本文对ecDNA在肿瘤细胞中的研究现状及其潜在意义作一综述。

## Abstract (en)

Extrachromosomal DNA (ecDNA) is a small segment of circular DNA located outside the chromosome, which has the function of self-replication. Recently, amplification of oncogenes on ecDNA has been proved to be a common phenomenon in tumor cells, and has some characteristics worth studying, such as correlation with patients' poor prognosis. Multiple chromosomal events are involved in the formation of ecDNA, and its amplification can directly increase the number of DNA copies of extra-chromosomal oncogenes and accelerate the generation and development of tumors. Moreover, the segregation pattern of unequal transmission of parental ecDNA cells to offspring not only increases tumor heterogeneity, but also enhances tumor adaptation to environment and response to therapy. This article reviews the current status and potential significance of ecDNA in tumor cells.

肿瘤发生过程中，癌基因扩增是常见的细胞改变之一。癌基因^[1]^和功能元件如增强子^[2]^的过表达，能给细胞提供选择性生长优势。研究^[4]^表明，肿瘤细胞中可携带癌基因的染色体外DNA（extrachromosomal DNA, ecDNA）是癌基因大量扩增的直接原因^[3]^，可以增加肿瘤遗传异质性。ecDNA也已被证实在肿瘤细胞中普遍存在^[5]^，且患者携带ecDNA往往提示更差的预后^[6]^。本综述将重点阐述ecDNA基本特征和其与肿瘤发生发展的关系。

## 1 染色体外DNA基本概念与特征

1965年染色体外DNA被首次发现，研究人员在哺乳动物DNA样本中观察到大小不一的DNA圆环，大的圆环常两两成对，故被称为双微钟（double minutes, DMs）^[7, 8]^。使用现代技术研究表明，DMs存在于各种类型的癌细胞中^[9, 10]^，并含有原癌基因^[5, 11-14]^。它的基本特征是一种在光学显微镜下可见的大型的（1 Mb-3 Mb或以上）、具有一个或多个完整基因和调控区的环状DNA，并且缺乏着丝粒或端粒。且进一步研究^[5]^发现，肿瘤细胞中只有约30%的染色体外DNA是成对的DMs，故现在多将双微钟及其单体形式称为ecDNA，不再用曾经的DMs描述它们。

广义的染色体外DNA大小不一，从几百碱基对的microDNA^[15]^到几百kb的DMs^[11]^，广泛存在于秀丽隐杆线虫、酵母、果蝇、小鼠、人体正常细胞和癌细胞等多种生物体内^[16-22]^，一般被称为染色体外环状DNA（extrachromosomal circular DNA, eccDNA），不是本文的主要讨论重点。ecDNA属于eccDNA的一种，但eccDNA广泛存在，而ecDNA多见于肿瘤细胞内。

ecDNA的存在在癌症中很常见。对来自不同患者的肿瘤细胞系和永生非癌细胞系等进行量化ecDNA水平研究发现，ecDNA几乎不存在于正常细胞中，但在40%的各类型癌症细胞和90%患者脑部肿瘤移植模型中存在。且ecDNA含量细胞间差异很大，具有肿瘤特异性^[5, 23]^。其中以脑胶质母细胞瘤及骨肉瘤最常见，在约50%的上述肿瘤细胞中都能检测到ecDNA。肺癌、乳腺癌、胃癌和食管癌等肿瘤中较常见，而白血病、淋巴瘤、前列腺癌等中较为罕见^[6]^。

Wu等^[3]^利用二代测序、双色荧光原位杂交技术（fluorescence *in situ* hybridization, FISH）等方法，分析了60个常见的肿瘤细胞和临床样本，进一步证明ecDNA为环状结构且能够直接、大量产生含癌基因mRNA的特点。利用免疫荧光、ATAC-seq和MNase-seq技术对其的进一步研究显示，ecDNA不仅含有癌基因和调控序列，还在相应位置（如启动子）上含有活跃型组蛋白修饰，并能够形成核小体。ecDNA拥有比染色体DNA开放性更强的染色质，且这种特点由ecDNA本身性质决定，不依赖于染色体DNA。ecDNA还可以形成拓扑相关结构域，产生DNA超远距离相互作用，以调控基因表达^[3]^。上述结论也已通过数据库在临床大样本中得到了进一步的验证^[6]^。

## 2 ecDNA在肿瘤细胞中的产生机制

ecDNA在肿瘤细胞中的形成、维持、复制、消除等机制尚不清楚，以下都是基于实验现象提出的可能猜想与推断。目前得到较多认可的ecDNA形成模型如下所述：胞内先产生双键断裂、染色体重排或其他染色体异常事件，使DNA片段环化形成ecDNA。接着在丧失肿瘤抑制因子的情况下，可能绕过细胞衰老反应发生ecDNA复制，进而导致ecDNA拷贝数的迅速累积^[24]^。

染色体碎裂和附加体形成都可能导致ecDNA的形成。染色体碎裂涉及一个或多个染色体断裂和多重重排^[25]^。染色体碎裂导致的染色体端粒融合参与胶质母细胞瘤和小细胞肺癌中的ecDNA发生^[26, 27]^。对急性髓性白血病中含*MYC*基因的ecDNA结构研究^[28, 29]^发现，不同的ecDNA可以在相同的白血病细胞群中共存，提示其逐步进化而非单一起源的产生过程。诱导DLD-1细胞系中染色体分离错误能观察到一系列染色体间和染色体内基因组重排。同时还观察到染色体碎裂直接导致ecDNA形成，进而导致ecDNA上所含基因的扩增^[30]^。一项对结直肠癌细胞的研究^[31]^证明双链断裂能诱导ecDNA聚合，同时发现聚合发生在S期，提示同源重组可能参与其中。研究者以此提出了附加体模型。还有一种与断裂-融合-桥循环（breakage-fusion-bridge, BFB）周期相关的模型，也被认为与ecDNA产生相关^[32]^。几种ecDNA产生的可能机制如图 1所示。

**图 1:**
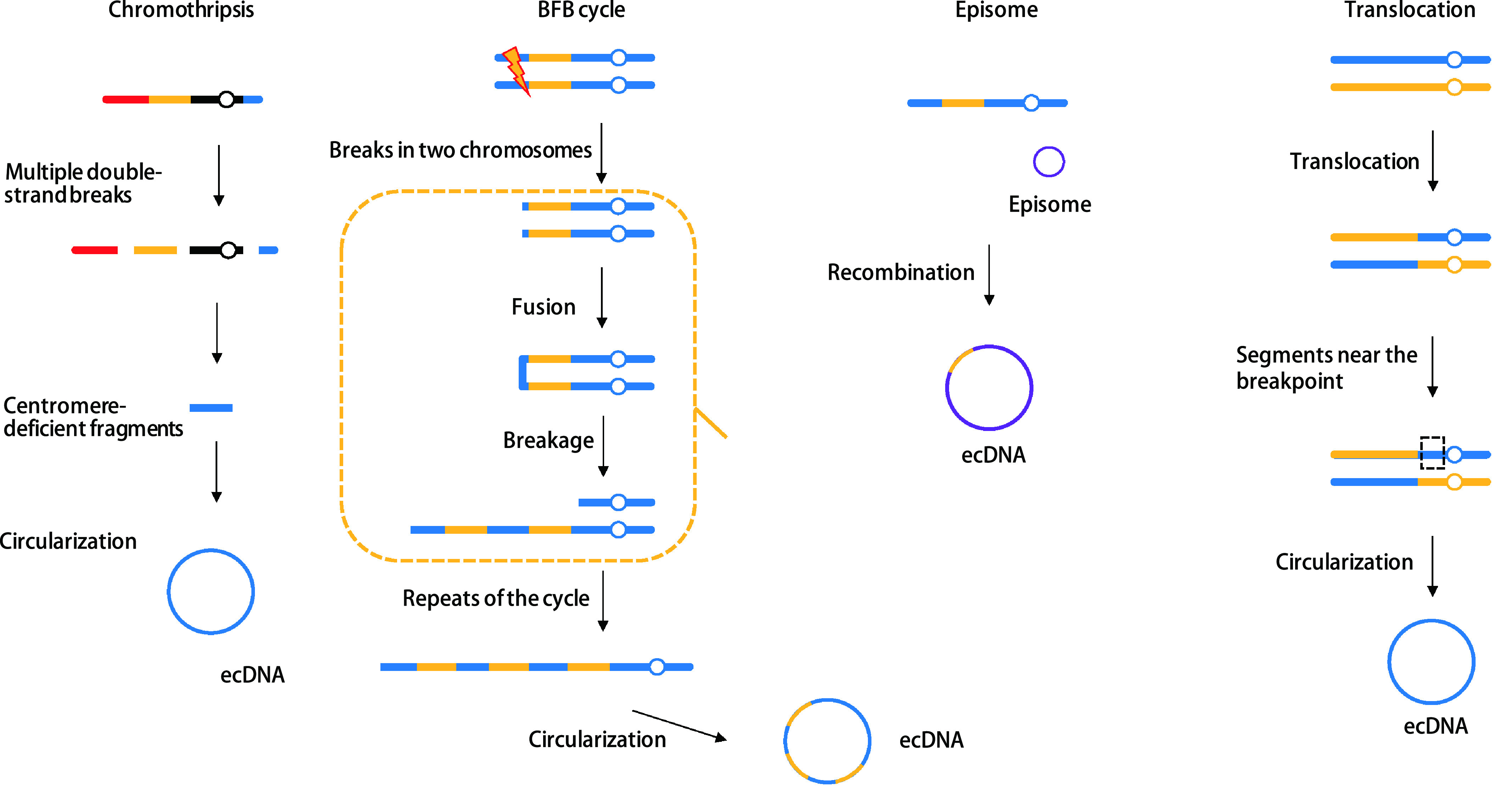
几种ecDNA可能的产生机制 Models of how ecDNA is formed. BFB: breakage-fusion-bridge; ecDNA: extrachromosomal DNA.

已经观察到ecDNA扩增子的形成与染色体不稳定及DNA缺陷修复机制相关。有研究^[33-35]^通过引入包含哺乳动物复制起始区和核基质附着区的质粒，成功扩增了ecDNA并对其形成过程进行描述，即含哺乳动物复制起始区（initiation region, IR）/基质附着区（matrix attachment region, MAR）质粒开始复制为小的染色体环，再融合形成较大结构。

不同的信号通路可以参与诱导含有不同癌基因的ecDNA形成。PI3K/Akt/BRCA1-Abraxas通路参与了SEI1诱导的ecDNA的形成，激活和抑制该通路可以分别导致小鼠体内ecDNA数量增加和减少^[36]^。抑制MAPK信号转导通路中ERK1/2的激活能显著减少ecDNA的数量及其携带基因的扩增和表达程度^[37]^。

## 3 ecDNA与肿瘤发生发展

ecDNA通过多种途径帮助细胞获得竞争优势。ecDNA在随机位点上形成的过程中，含有致癌基因和调控元件的位点被优先选择。ecDNA传代过程中环状扩增子的不均等分离，增加了子代细胞ecDNA的表达，这种表达不受基因剂量影响，能够帮助癌基因进一步表达，并使拷贝数快速增加^[38]^。ecDNA的重新整合也能帮助细胞获得更多选择优势。一项对扩增子序列重新整合到*DCLK1*基因位点中的研究^[39]^表明该序列导致杂合性丢失，随后肿瘤抑制功能丧失。

### 3.1 ecDNA与肿瘤细胞癌基因扩增

一项对3, 212例癌症患者全基因组测序数据分析的结果^[6]^证明，许多重要的原癌基因主要以ecDNA为载体进行扩增，如*MDM2*、*MYC*、表皮生长因子受体（epidermal growth factor receptor, *EGFR*）、*CDK4*、*ERBB2*等经典基因，表明它与肿瘤细胞癌基因拷贝数增加和基因转录水平提高相关^[3]^。同时，也发现了多个携带不同癌基因（*MYC*和*EGFR*）的ecDNA^[4]^。

为了探究ecDNA与癌基因扩增的关系，Turner等^[5]^开发出一种无偏差图像及计算机分析方法用与检测胞外基因，发现所有癌基因的完全性扩增或者于ecDNA单独完成，或者与ecDNA及染色体同时进行。这种ecDNA相关的扩增将大大增强肿瘤异质性，使基因拷贝数快速到达并维持在较高水平。并且基因扩增在肿瘤体内和体外生长过程中都被保留。在对胶质母细胞瘤的一项研究^[4]^中，*MYC*、*MYCN*、*EGFR*、*MET*等基因都能观察到这一现象。

对神经母细胞瘤基因组研究^[39]^发现，大多数染色体内部和之间的基因重排，与ecDNA区域相符合，表明染色体间的重组位点能在环状位置形成树状形式，推测ecDNA可以重新整合到线性基因组中形成嵌合体，促使癌基因重塑。这些事件在帮助肿瘤基因扩增的同时，也有助于肿瘤基因组模式化。

去除肿瘤细胞核DNA能使扩增癌基因丢失^[33]^。对人类早幼粒细胞白血病HL-60细胞系研究^[40-42]^发现，肿瘤细胞的自发分化涉及c-MYC拷贝数主动清除，并且在羟基脲存在下，于有丝分裂形成富含ecDNA的微核后，ecDNA的丢失加速。

### 3.2 ecDNA与肿瘤细胞均匀染色区（homogeneously staing regien, HSR）

ecDNA可以动态整合到异常基因组位置，如HSR上^[5, 23]^。HSR是肿瘤细胞g显带标本上染色均匀的区域，与基因重复扩增相关，为癌细胞的一种特有结构，含有癌基因。GBM39细胞系中ecDNA导致的耐药机制之一就是ecDNA在HSR上的可逆重定位^[43]^。

一项对异种移植的人少突胶质细胞瘤的研究^[44]^发现，*EGFR*和*MYC*基因座共扩增在传代早期以ecDNA形式出现，晚期则以HSR形式出现。这种转变的过程中，扩增区域有多次重排和缺失。两种扩增形式中，普遍存在的是非同源末端连接和微同源介导的末端连接。ecDNA在连续传代过程中作为HSR重新定位，并积累额外断点簇。研究^[44]^还发现，一些片段不是随机融合，而是由可能的远端DNA序列相互作用时，相邻断点的伴随修复导致。

在对结直肠癌细胞中ecDNA和HSR抗性HT-29细胞系的同源重组活性检测实验^[45]^中，发现该细胞系比氨甲蝶呤敏感细胞有更活跃的同源重组活性。通过沉默BRCA1抑制同源重组（homologous recombination, HR）活性能够减少ecDNA及ecDNA来源的扩增基因拷贝数，且该变化随细胞周期和MTX敏感性增加而增加。但上述现象在HSR抗性细胞中没有被观察到。这表明HR途径在染色体外和染色体内基因扩增中起着不同作用。同一细胞系的研究中，抑制非同源末端连接相关关键基因，也能观察到ecDNA的减少^[46]^。提示HR通路可能是针对染色外扩增的新靶点。

### 3.3 ecDNA与增强子

ecDNA能使得癌基因利用增强子增强其转录能力。ecDNA相比线性DNA，核小体结构不紧密，开放性强，能够实现超远距离染色质接触，能够与调控元素产生远距离相互作用^[3]^。在一项研究^[3]^中，5种不同实体肿瘤中都观察到，癌基因和其附近增强子通过ecDNA形式共同扩增。这种情况在神经母细胞瘤及Wilms肿瘤中MYCN和髓母细胞瘤中MYC基因中最为显著。并且癌基因不仅能通过ecDNA上的内源增强子进行扩增，还可以从其他拓扑相关域中选择调控元件。一项研究^[47]^中ecDNA的EGFR1扩增体被观察到经历了增强子重组，内源和异位增强子被合并至每个能增强细胞适应性的增强子中。Helmsauer和他的同事^[48]^观察到神经母细胞瘤细胞系中MYCN扩增子缺乏内源性增强子，从同一远端区域劫持异位增强子进入ecDNA结构。

### 3.4 ecDNA与基因组重排

对神经母细胞瘤的研究^[39]^意外发现，ecDNA还参与体细胞基因组重排，它可通过嵌合体循环、重新整合进入线性基因组等方式，促进肿瘤基因组重塑。肿瘤进展也可能引起ecDNA重排，与临床不良预后相关。ecDNA的多点突变对肿瘤基因组重塑有重要的意义。在1例复发的胶质母细胞瘤中，ecDNA在KIT/PDGFRA/PI3K/mTOR轴上渐进进化。最终，包含PDGFRA、KIT和CDK4的ecDNA以相互排斥的方式，取代原发肿瘤中异柠檬酸脱氢酶1（isocitrate dehydrogenase I, *IDH1*）的突变优势，使得复发肿瘤呈现出新的克隆突变表型^[35]^。

### 3.5 ecDNA与肿瘤耐药性

#### 3.5.1 ecDNA拷贝数动态变化调节癌基因表达

环境对细胞癌基因数量改变也可能通过ecDNA变化水平反映。ecDNA和细胞外基质在培养过程中被癌细胞丢失，并且野生型和突变型ecDNA可能在同一肿瘤中共存^[49]^，表明包含ecDNA的肿瘤细胞有快速适应环境变化的能力。

这种ecDNA数量动态变化也与肿瘤耐药性相关。在胶质母细胞瘤患者临床样本和单细胞分析患者的衍生模型中，应用EGFR酪氨酸激酶抑制剂时，细胞能够动态调节突变型*EGFR*的表达，使自身到达生长平衡的最佳状态。其中对抑制剂抗性通过从ecDNA中消除EGFR实现。停药后，*EGFR*突变能够可逆地重新在ecDNA上出现，表明癌细胞可以通过这种途径逃避针对ecDNA的治疗^[43]^。相似的是，癌症药物吉西他滨能导致卵巢癌细胞株UACC-1598内ecDNA丢失^[50]^。在对人类结直肠癌细胞系NCI-H716和人类恶性原始神经外胚层肿瘤细胞系SK-PN-DW研究中发现，抑制癌基因在ecDNA上表达，可以导致ecDNA数量减少，并使细胞增殖和侵袭能力也一定程度降低^[51]^。对GBM39细胞系*EGFR*vIII基因扩增的分析^[5, 43]^证明，厄洛替尼使ecDNA重组为HSRs。去除药物后，ecDNA扩增物重新出现。

在一项经典研究^[52]^中，用甲氨蝶呤处理结直肠癌细胞HT-29，可观察到位于ecDNA片段上的二氢叶酸还原酶（dihydrofolatereductase, *DHFR*）基因拷贝数增加，克服了甲氨蝶呤对叶酸代谢抑制性作用，使肿瘤产生耐药性。

胶质母细胞瘤具有群体异质性。将*EGFR*突变体高表达和低表达的细胞分离分别体外培养一段时间又会变成混杂表达群体。使用表皮生长因子受体抑制剂，能够在细胞、小鼠、患者体内都观察到高表达细胞减少，而低表达细胞存活。药物撤除后，*EGFR*高拷贝数细胞重新出现，异质性恢复。这种现象依赖于ecDNA在细胞中期对*EGFR*VIII基因拷贝数的调节机制，而*EGFR*VIII基因几乎完全在ecDNA上被扩增。这种广泛的胞外不稳定性，使肿瘤逃避药物攻击，增加耐药性^[43]^。

#### 3.5.2 ecDNA增加肿瘤细胞异质性

ecDNA驱动的拷贝数扩增可使肿瘤细胞增加瘤内基因异质性，而ecDNA在子代细胞中的不均等分离则能够增加细胞多样性，使其迅速适应环境压力。

上文已经提及，ecDNA对于一个细胞群体来说数量分布差异很大^[5]^。用体细胞单核苷酸变异体标记的肿瘤细胞和拥有ecDNA的亚克隆进化是不同的，进一步证明ecDNA与染色体有不同的遗传模式。根据ecDNA特点可以推断，肿瘤分裂过程中，因为缺乏着丝粒，ecDNA不能均匀分配入后代细胞，或者通过不同水平、不同类型的ecDNA的不均等扩增^[53]^，增加肿瘤异质性，加速肿瘤进化。

ecDNA还可以在次生体细胞突变之后形成。一个包含多个ecDNA拷贝的细胞中每个拷贝都可能在复制中的同一基因组区域获得不同突变而加速ecDNA进化。胶质母细胞瘤原发和复发样本中，都发现了包括相同致癌基因的新的ecDNA。不同的ecDNA相互竞争、演化，会迅速增加肿瘤异质性^[54]^。

有研究^[46]^已经着眼于探究通过减少染色体外基因扩增改善肿瘤药物治疗疗效。非同源性末端接合（non-homologous end joining, NHEJ）蛋白在含有ecDNA的MTX耐药结直肠癌细胞中表达增加，抑制或缺失非同源性末端接合蛋白DNA-PKcs能减少*DHFR*基因扩增，使ecDNA消失，增加细胞MTX敏感性，提示NHEJ可能作为MTX耐药结直肠癌靶点。通过沉默*BRCA1*来抑制HR活性也可以增加耐药肿瘤细胞对MTX的敏感性^[55]^。

以上结果显示，拥有ecDNA的癌细胞可以更快地应对环境的各种变化，这也给肿瘤治疗带来新的挑战^[56, 57]^。治疗过程中所接受的化学疗法、放射疗法和药物靶向疗法等，都可能影响ecDNA拷贝数而增加肿瘤细胞逃避免疫监视的能力^[58-60]^。未来需要研究ecDNA是否使肿瘤细胞更容易逃避治疗和免疫系统监控？如果是，可能的分子机制是怎样的？这些都有助于进一步研究更有效的治疗方案。

## 4 ecDNA作为生物标志物的潜力

近年来血液中循环DNA已经展现其作为生物标志物的巨大潜力，但是研究多着眼于线性DNA。目前有研究^[61]^发现肿瘤细胞会释放环状微DNA入血，并且更加稳定。另外一项对妊娠妇女血浆中染色体外环状DNA的研究^[62]^显示，胎儿可将eccDNA释放到孕妇血浆中。这些eccDNA来源于各种组织和细胞，正常与病变情况都有，已经显示出与疾病有相关性，提示其作为生物标志物潜在的用于疾病监测、治疗评估和肿瘤进展监测的价值^[63]^。此外，虽然ecDNA在急性骨髓性白血病等血液肿瘤和淋巴组织肿瘤中极为罕见，但仍有相关的病例报道^[64]^和利用ecDNA进行疾病监测的研究^[65]^，研究中使用FISH和g显带技术检测恶性肿瘤细胞数量变化，初诊检出率为1.23%（6/489），或许可以给我们提供一些新思路。这些研究都提示我们，环状DNA结构或许可以更加稳定地在血液中存在，并且在以往的研究中常常被忽略。未来可以挖掘ecDNA在分子标志物、肿瘤转移研究等方面的潜力。ecDNA不仅能够应用于肿瘤预后和诊断，还能了解肿瘤起源。也有研究者认为ecDNA可能是ctDNA和cfDNA的起源，相关研究有待进一步开展。

## 5 总结与展望

虽然染色体外DNA早就被发现，但一直被研究者们忽视。通常使用高通量短读长DNA测序方法推断肿瘤DNA拷贝数分布时，扩增基因匹配到对应染色体上导致ecDNA可能被大量丢失以致被忽略。随着越来越多实验证明ecDNA的普遍性和其在加速肿瘤细胞进化中的重要意义，我们有必要重新考虑这些新的基因放大途径对于肿瘤治疗的意义。目前还有很多问题需要解决：ecDNA的产生、维持和消除机制是什么？我们能否从自然界其他类似的圆形DNA（如质粒）的特征中类比ecDNA的性质？ecDNA为肿瘤带来的特异性特征是否可以被用于癌症治疗？环境的压力又会对ecDNA产生哪些影响？ecDNA作为生物标志物又有哪些潜力？认识到扩增癌基因的位置、空间结构和特点，对癌症的产生、进展和治疗相关研究都有重要的意义。

## References

[1] Beroukhim R, Mermel CH, Porter D (2010). The landscape of somatic copy-number alteration across human cancers. Nature.

[2] Weischenfeldt J, Dubash T, Drainas AP (2017). Pan-cancer analysis of somatic copy-number alterations implicates IRS4 and IGF2 in enhancer hijacking. Nat Genet.

[3] Wu S, Turner KM, Nguyen N (2019). Circular ecDNA promotes accessible chromatin and high oncogene expression. Nature.

[4] deCarvalho AC, Kim H, Poisson LM (2018). Discordant inheritance of chromosomal and extrachromosomal DNA elements contributes to dynamic disease evolution in glioblastoma. Nat Genet.

[5] Turner KM, Deshpande V, Beyter D (2017). Extrachromosomal oncogene amplification drives tumour evolution and genetic heterogeneity. Nature.

[6] Kim H, Nguyen NP, Turner K (2020). Extrachromosomal DNA is associated with oncogene amplification and poor outcome across multiple cancers. Nat Genet.

[7] Hotta Y, Bassel A (1965). Molecular size and circularity of dna in cells of mammals and higher plants. Proc Natl Acad Sci U S A.

[8] Cox D, Yuncken C, Spriggs AI (1965). Minute chromatin bodies in malignant tumours of childhood. Lancet.

[9] Fan Y, Mao R, Lv H (2011). Frequency of double minute chromosomes and combined cytogenetic abnormalities and their characteristics. J Appl Genet.

[10] Stark GR, Debatisse M, Giulotto E (1989). Recent progress in understanding mechanisms of mammalian DNA amplification. Cell.

[11] Vogt N, Lefèvre SH, Apiou F (2004). Molecular structure of double-minute chromosomes bearing amplified copies of the epidermal growth factor receptor gene in gliomas. Proc Natl Acad Sci U S A.

[12] Storlazzi CT, Lonoce A, Guastadisegni MC (2010). Gene amplification as double minutes or homogeneously staining regions in solid tumors: origin and structure. Genome Res.

[13] Del Rey J, Prat E, Ponsa I (2010). Centrosome clustering and cyclin D1 gene amplification in double minutes are common events in chromosomal unstable bladder tumors. BMC Cancer.

[14] Zuberi L, Adeyinka A, Kuriakose P (2010). Rapid response to induction in a case of acute promyelocytic leukemia with *MYC* amplification on double minutes at diagnosis. Cancer Genet.

[15] Shibata Y, Kumar P, Layer R (2012). Extrachromosomal microDNAs and chromosomal microdeletions in normal tissues. Science.

[16] Cohen S, Méchali M (2002). Formation of extrachromosomal circles from telomeric DNA in Xenopus laevis. EMBO Rep.

[17] Møller HD, Parsons L, Jørgensen TS (2015). Extrachromosomal circular DNA is common in yeast. Proc Natl Acad Sci U S A.

[18] Stanfield SW, Lengyel JA (1979). Small circular DNA of Drosophila melanogaster: chromosomal homology and kinetic complexity. Proc Natl Acad Sci U S A.

[19] Flores SC, Moore TK, Gaubatz JW (1987). Dispersed repetitive sequences of the mouse genome are differentially represented in extrachromosomal circular DNAs *in vivo*. Plasmid.

[20] Cohen S, Yacobi K, Segal D (2003). Extrachromosomal circular DNA of tandemly repeated genomic sequences in Drosophila. Genome Res.

[21] Shoura MJ, Gabdank I, Hansen L (2017). Intricate and cell type-specific populations of endogenous circular DNA (eccDNA) in *Caenorhabditis elegans* and *Homo sapiens*. G3 (Bethesda).

[22] Møller HD, Mohiyuddin M, Prada-Luengo I (2018). Circular DNA elements of chromosomal origin are common in healthy human somatic tissue. Nat Commun.

[23] Sanborn JZ, Salama SR, Grifford M (2013). Double minute chromosomes in glioblastoma multiforme are revealed by precise reconstruction of oncogenic amplicons. Cancer Res.

[24] Verhaak RGW, Bafna V, Mischel PS (2019). Extrachromosomal oncogene amplification in tumour pathogenesis and evolution. Nat Rev Cancer.

[25] Stephens PJ, Greenman CD, Fu B (2011). Massive genomic rearrangement acquired in a single catastrophic event during cancer development. Cell.

[26] Cortés-Ciriano I, Lee JJ, Xi R (2020). Comprehensive analysis of chromothripsis in 2, 658 human cancers using whole-genome sequencing. Nat Genet.

[27] Ratnaparkhe M, Wong JKL, Wei PC (2018). Defective DNA damage repair leads to frequent catastrophic genomic events in murine and human tumors. Nat Commun.

[28] Abbate AL, Tolomeo D, Cifola I (2018). MYC-containing amplicons in acute myeloid leukemia: genomic structures, evolution, and transcriptional consequences. Leukemia.

[29] Huh YO, Tang G, Talwalkar SS (2016). Double minute chromosomes in acute myeloid leukemia, myelodysplastic syndromes, and chronic myelomonocytic leukemia are associated with micronuclei, *MYC* or *MLL* amplification, and complex karyotype. Cancer Genet.

[30] Ly P, Brunner SF, Shoshani O (2019). Chromosome segregation errors generate a diverse spectrum of simple and complex genomic rearrangements. Nat Genet.

[31] Oobatake Y, Shimizu N (2020). Double-strand breakage in the extrachromosomal double minutes triggers their aggregation in the nucleus, micronucleation, and morphological transformation. Genes Chromosomes Cancer.

[32] Tanaka H, Watanabe T (2020). Mechanisms underlying recurrent genomic amplification in human cancers. Trends Cancer.

[33] Shimizu N (2009). Extrachromosomal double minutes and chromosomal homogeneously staining regions as probes for chromosome research. Cytogenet Genome Res.

[34] Shimizu N, Miura Y, Sakamoto Y (2001). Plasmids with a mammalian replication origin and a matrix attachment region initiate the event similar to gene amplification. Cancer Res.

[35] Shimizu N, Misaka N, Utani K (2007). Nonselective DNA damage induced by a replication inhibitor results in the selective elimination of extrachromosomal double minutes from human cancer cells. Genes Chromosomes Cancer.

[36] Tian X, Liu C, Wang X (2018). Sei-1 promotes double minute chromosomes formation through activation of the PI3K/Akt/BRCA1-Abraxas pathway and induces double-strand breaks in NIH-3T3 fibroblasts. Cell Death Dis.

[37] Sun W, Quan C, Huang Y (2015). Constitutive ERK1/2 activation contributes to production of double minute chromosomes in tumour cells. J Pathol.

[38] Kim H, Nguyen N, Turner K (2019). Frequent extrachromosomal oncogene amplification drives aggressive tumors. bioRxiv.

[39] Koche RP, Rodriguez-Fos E, Helmsauer K (2020). Extrachromosomal circular DNA drives oncogenic genome remodeling in neuroblastoma. Nat Genet.

[40] Shimizu N, Nakamura H, Kadota T (1994). Loss of amplified *c-myc* genes in the spontaneously differentiated HL-60 cells. Cancer Res.

[41] Eckhardt SG, Dai A, Davidson KK (1994). Induction of differentiation in HL60 cells by the reduction of extrachromosomally amplified c-myc. Proc Natl Acad Sci U S A.

[42] Valent A, Bénard J, Clausse B (2001). *In vivo* elimination of acentric double minutes containing amplified *MYCN* from neuroblastoma tumor cells through the formation of micronuclei. Am J Pathol.

[43] Nathanson DA, Gini B, Mottahedeh J (2014). Targeted therapy resistance mediated by dynamic regulation of extrachromosomal mutant *EGFR* DNA. Science.

[44] Vogt N, Gibaud A, Lemoine F (2014). Amplicon rearrangements during the extrachromosomal and intrachromosomal amplification process in a glioma. Nucleic Acids Res.

[45] Cai M, Zhang H, Hou L (2019). Inhibiting homologous recombination decreases extrachromosomal amplification but has no effect on intrachromosomal amplification in methotrexate-resistant colon cancer cells. Int J Cancer.

[46] Meng X, Qi X, Guo H (2015). Novel role for non-homologous end joining in the formation of double minutes in methotrexate-resistant colon cancer cells. J Med Genet.

[47] Morton AR, Dogan-Artun N, Faber ZJ (2019). Functional enhancers shape extrachromosomal oncogene amplifications. Cell.

[48] Helmsauer K, Valieva M, Ali S (2019). Enhancer hijacking determines intra- and extrachromosomal circular MYCN amplicon architecture in neuroblastoma. bioRxiv.

[49] Nikolaev S, Santoni F, Garieri M (2014). Extrachromosomal driver mutations in glioblastoma and low-grade glioma. Nat Commun.

[50] Yu L, Zhao Y, Quan C (2013). Gemcitabine eliminates double minute chromosomes from human ovarian cancer cells. PLoS One.

[51] Ji W, Bian Z, Yu Y (2014). Expulsion of micronuclei containing amplified genes contributes to a decrease in double minute chromosomes from malignant tumor cells. Int J Cancer.

[52] Morales C, Ribas M, Aiza G (2005). Genetic determinants of methotrexate responsiveness and resistance in colon cancer cells. Oncogene.

[53] Koduru P, Chen W, Haley B (2019). Cytogenomic characterization of double minute heterogeneity in therapy related acute myeloid leukemia. Cancer Genet.

[54] Xu K, Ding L, Chang TC (2019). Structure and evolution of double minutes in diagnosis and relapse brain tumors. Acta Neuropathol.

[55] Cai M, Zhang H, Hou L (2019). Inhibiting homologous recombination decreases extrachromosomal amplification but has no effect on intrachromosomal amplification in methotrexate-resistant colon cancer cells. Int J Cancer.

[56] Xue Y, Martelotto L, Baslan T (2017). An approach to suppress the evolution of resistance in *BRAF*(V600E)-mutant cancer. Nat Med.

[57] Nathanson DA, Gini B, Mottahedeh J (2014). Targeted therapy resistance mediated by dynamic regulation of extrachromosomal mutant *EGFR* DNA. Science.

[58] Zhang AW, McPherson A, Milne K (2018). Interfaces of malignant and immunologic clonal dynamics in ovarian cancer. Cell.

[59] Grasso CS, Giannakis M, Wells DK (2018). Genetic mechanisms of immune evasion in colorectal cancer. Cancer Discov.

[60] McGranahan N, Rosenthal R, Hiley CT (2017). Allele-specific HLA loss and immune escape in lung cancer evolution. Cell.

[61] Kumar P, Dillon LW, Shibata Y (2017). Normal and cancerous tissues release extrachromosomal circular DNA (eccDNA) into the circulation. Mol Cancer Res.

[62] Sin STK, Jiang P, Deng J (2020). Identification and characterization of extrachromosomal circular DNA in maternal plasma. Proc Natl Acad Sci U S A.

[63] Zhu J, Chen S, Zhang F (2018). Cell-free eccDNAs: a new type of nucleic acid component for liquid biopsy?. Mol Diagn Ther.

[64] Nguyen JC, Kubik MJ, Broome HE (2015). Successful treatment of both double minute of *C-MYC* and *BCL-2* rearrangement containing large B-cell lymphoma with subsequent unfortunate development of therapy-related acute myeloid leukemia with t(3;3)(q26.2;q21). Pathol Res Pract.

[65] Jeon Y, Kim SY, Kim M (2014). Fluorescence *in situ* hybridization panel for monitoring of minimal residual disease in patients with double minute chromosomes. Blood Cells Mol Dis.

